# Microcystic, elongated, and fragmented pattern of invasion in relation to histopathologic and clinical prognostic factors in endometrioid endometrial adenocarcinoma

**DOI:** 10.4274/jtgga.2017.0016

**Published:** 2017-09-01

**Authors:** M. Murat Naki, Gülbin Oran, Seza Ümit Tetikkurt, Cavide Fatma Sönmez, İlknur Türkmen, Faruk Köse

**Affiliations:** 1 Department of Obstetrics and Gynecology, Acıbadem University Faculty of Medicine, İstanbul, Turkey; 2 Department of Pathology, Medipol University Faculty of Medicine, İstanbul, Turkey; 3 Department of Pathology, Bağcılar Training and Research Hospital, İstanbul, Turkey; 4 Department of Pathology, Bezmialem Vakıf University Faculty of Medicine, İstanbul, Turkey

**Keywords:** Endometrioid endometrial adenocarcinoma, microcystic, elongated, fragmented pattern, tumor grade, myometrial invasion, lymphovascular space invasion

## Abstract

**Objective:**

To investigate the association of microcystic, elongated, and fragmented (MELF) pattern of invasion with prognostic factors in endometrioid endometrial adenocarcinoma (EEA).

**Material and Methods:**

Stained tissue sections from 83 cases of EEA operated by the same gynecologic oncologist were reviewed to identify cases showing MELF-type invasion in this retrospective study. The association of MELF pattern with age, tumor grade, depth of myometrial invasion, and presence of lymphovascular space invasion (LVSI) was analyzed.

**Results:**

FIGO grade 2 and grade 1 tumors were evident in 53.0% and 38.6% of patients, respectively. Depth of myometrial invasion was <50% in 72.0% of patients, and LVSI was absent in 77.1%. MELF pattern was confirmed in 35 (42.2%) patients. Presence of MELF pattern was associated with significantly higher mean ± standard deviation age (62.9±6.9) years vs. 58.9±9.1 years, p=0.033), and found to be more likely in patients with high-grade tumor (FIGO grade III; 85.7% vs. 14.3%, p<0.001), deep (≥50%) myometrial invasion (78.3% vs. 21.7%, p<0.001), and presence of LVSI (94.7% vs. 5.3%, p<0.001) as compared with absence of MELF pattern.

**Conclusion:**

In conclusion, our findings revealed a high rate of MELF pattern among patients with EEA alongside the association of MELF pattern with poor prognostic factors such as high grade tumor, deep myometrial invasion, and LVSI.

## INTRODUCTION

Endometrioid endometrial adenocarcinoma (EEA) has a favorable prognosis with overall 5-year survival rates of 90%, reaching 93-94% for International Federation of Gynecology and Obstetrics (FIGO) grade 1 and 2 tumors (1, 2).

Besides the increased risk of extra-uterine spread of tumor with the depth of myometrial invasion, certain patterns of myometrial invasion have also been proposed as a potential prognostic factors in endometrial carcinomas, which may play a role in predicting the pattern of lymph node involvement as well as metastasis (3, 4, 5, 6, 7).

The microcystic, elongated, and fragmented (MELF) pattern is a distinctive presentation of myometrial invasion with unusual changes to neoplastic glands, which was first described by Murray et al. (8) in 2003. Although it was initially thought to represent a simple degenerative process, with identification of the restriction of MELF pattern invasion to myoinvasive low-grade carcinomas of endometrioid type, these changes have been suggested to be an active cellular process indicating a specific tumor-stromal interaction (4, 5).

Tumors with MELF pattern have been associated with deposits of carcinoma in lymph nodes that resemble hystiocytes, which forms the basis of application of keratin stains to identify single tumor cells in lymph node sinuses, as well as experimentally in the setting of sentinel lymph node evaluation (9). Nonetheless, despite the documented association of MELF pattern with lymphovascular space invasion (LVSI) and lymph node metastasis, it remains uncertain as to whether presence of MELF pattern invasion has clinical significance in EEA (5, 6, 7, 8, 10). Our study was therefore designed to study and examine the frequency of MELF pattern invasion in patients with EEA and to determine its association with clinical and histopathologic prognostic factors.

## MATERIAL AND METHODS

Stained tissue samples from 83 cases of operated EEA were reviewed to identify cases showing MELF-type invasion in this retrospective study. The inclusion criterion was endometrioid-type endometrial adenocarcinoma. Non-endometrioid-type adenocarcinoma was excluded. The study was conducted in full accordance with local GCP guidelines and current legislation, and our institutional ethics committee gave permission to use patient data for publication (19.10.2016/487).

Data on age, tumor grade, depth of myometrial invasion and LVSI were recorded for each patient. Tumor samples were re-evaluated by the same pathologist to investigate MELF pattern. The association of MELF pattern with age, tumor grade, depth of myometrial invasion, and presence of LVSI was analyzed. Tissue samples were graded using the grading system identified by FIGO: a solid non-squamous content up to 5% was considered grade 1, 6 to 50% as grade 2, and >50% as grade 3 (11). Hysterectomy specimens stained with hematoxylin and eosin (H&E) were evaluated to identify MELF pattern exhibition and MELF-positive cases as previously described by Murray et al. (8). LVSI tumor fragments present in the endothelium-lined vascular/lymphatic spaces, either in the tumor or distant from it, were also evaluated in H&E stained specimens with or without MELF-expression.

Depth of myometrial invasion was determined based on the tumor invading the myometrium down to deepest margin and lying over the endomyometrial junction and classified as invasion through <50% or ≥50% of the myometrial thickness.

### Statistical analysis

MedCalc Statistical Software version 12.7.7 (MedCalc Software BVBA, Ostend, Belgium; http://www.medcalc.org; 2013) was used for statistical analysis. The Chi-square (χ^2^) test and Fisher’s exact test were used to compare categorical data, and numeric data were analyzed using Student’s t-test for independent variables with normal distribution. Data are expressed as mean ± standard deviation (SD), minimum-maximum and percentage (%) where appropriate. P<0.05 was considered statistically significant.

## RESULTS

FIGO grades 2, 1, and 3 tumors were evident in 53.0%, 38.6%, and 8.4% of patients, respectively. Depth of myometrial invasion was <50% in 72.0% of cases (stage IA), and LVSI was absent in 77.1%. MELF pattern invasion was confirmed in 35 (42.2%) patients (Table 1).

Presence of MELF pattern was associated with significantly higher mean age, 62.9±6.9 years vs. 58.9±9.1 years, p=0.033, and found to be more likely in cases of high-grade tumor (FIGO grade III; 85.7% vs. 14.3%, p<0.001), deep (≥50%) myometrial invasion (78.3% vs. 21.7%, p<0.001), and lymphovascular invasion (94.7% vs. 5.3%, p<0.001) as compared with absence of MELF pattern invasion (Table 1).

## DISCUSSION

Our findings revealed the presence of MELF pattern invasion in 42.2% of patients with EEA and greater likelihood of MELF positivity in older patients; the presence of factors indicates poor prognosis including deep myometrial invasion, high grade tumor, and LVSI.

The incidence of MELF in our cohort (42.2%) was higher than the incidence (23.1%) reported previously in Turkish patients (n=121) (7), but consistent with the upper level of the range (7 to 48%) for the overall incidence of MELF reported in different studies among patients with EEA (5, 7, 10, 12, 13). Our findings support the more frequent observation of deep myometrial invasion and LVSI in MELF-positive cases (7), unlike previous reports indicating the more common observation of MELF invasion in low-grade (FIGO grade 1 or 2) EEAs (5, 6, 7). The percentage of patients with and without MELF pattern was significantly higher in high-grade (FIGO grade 3) tumors in our cohort. Indeed, cervical stromal involvement, lymph nodal metastasis, and clinically advanced stage were reported to be more common in the presence of MELF pattern invasion, and MELF pattern positivity, as well as involvement of cervical stroma were shown to be independent determinants of lymph node metastasis (7).

In total, 18 of 19 patients with LVSI and 28 with >50% myometrial invasion (94.7% and 78.3%, respectively) had MELF pattern invasion in our study. This supports data from a past study, which indicated that MELF-positive cases were more likely to exhibit LVSI and ≥50% MI than MELF-negative cases (78.5% vs. 13.9% and 78.5% vs. 32.2%, respectively) (7). MELF pattern, when accompanied with fibromyxoid stromal response, was shown be related to LVSI and a worse long- term prognosis (8), and an invasive glandular morphology was suggested to be a stronger marker of LVSI risk than the depth of invasion (7).

High-grade histologic subtype, older age, myometrial invasion depth, and positivity of LVSI are well-established interacting adverse prognostic factors that are predictive of disease outcome in endometrial neoplasia (1, 2, 6, 14). Presence of LVI and MELF-type invasion were shown to be significantly associated with an increased likelihood of nodal metastasis (5). Thus, the increased likelihood of poor prognostic factors among MELF-positive cases in our cohort emphasizes the implications of accurate histologic assessment of myometrial invasion and recognition of MELF pattern invasion in clinical practice in terms of grading of EEAs, and assessing long-term prognosis given its association with LVSI, lymph node metastasis, and extra-uterine disease (4, 5, 7, 8, 10, 14). In addition, a past study that analyzed risk factors for recurrence in low-grade EEA showed that MELF myoinvasion pattern, deep myoinvasion, LVSI, and high-grade tumor in metastatic foci were factors that predicted increased recurrence risk at sites other than the vagina (5).

The likelihood of increased tumor progression and adverse prognosis in the presence of MELF pattern invasion in EEAs has been associated with enhanced dissemination of neoplastic cells due to a fibromyxoid stromal reaction creating a low resistance milieu, as well as a likelihood of transition of epithelial mesenchyme that allows surrounding stromal infiltration (4, 5, 8, 14, 15). Nevertheless, the clinical relevance of MELF pattern positivity in endometrial adenocarcinoma has not yet been elucidated given that the relation of MELF invasion to lymph node metastasis or poor prognosis was not confirmed in some studies (5, 6, 7, 8, 10).

In conclusion, our findings revealed a high rate of MELF pattern invasion among patients with EEC alongside the association of MELF pattern invasion with poor prognostic factors such as high-grade tumor, deep myometrial invasion, and lymphovascular invasion. Our findings emphasize the importance of accurate histologic assessment of myometrial invasion, and the likely role of identifying MELF pattern invasion with frozen section or probe curettage in configuring surgical treatment among patients with EEA. Nonetheless, larger scale studies are necessary to justify the consideration of MELF pattern invasion as a risk factor for higher-stage disease, lymph node metastasis, and thus poor outcomes in EEA.

## Figures and Tables

**Table 1:**
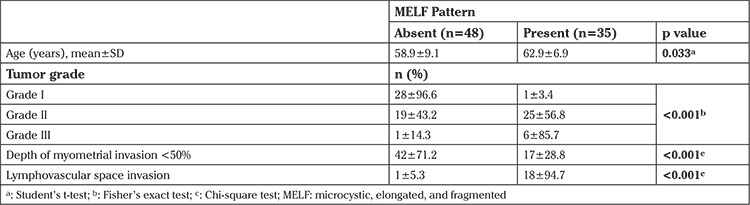
Prognostic factors in the overall study population with respect to MELF pattern invasion

## References

[1] Lewin SN, Herzog TJ, Barrena Medel NI, Deutsch I, Burke WM, Sun X, et al (2010). Comparative performance of the 2009 International Federation of Gynecology and Obstetrics' staging system for uterine corpus cancer. Obstet Gynecol.

[2] Kilgore JE, Jackson AL, Ko EM, Soper JT, Van Le L, Gehrig PA, et al (2013). Recurrence-free and 5-year survival following robotic-assisted surgical staging for endometrial carcinoma. Gynecol Oncol.

[3] Sorosky JI (2008). Endometrial cancer. Obstet Gynecol.

[4] Stewart CJ, Little L (2009). Immunophenotypic features of MELF pattern invasion in endometrial adenocarcinoma: evidence for epithelial-mesenchymal transition. Histopathology.

[5] Pavlakis K, Messini I, Vrekoussis T, Panoskaltsis T, Chrysanthakis D, Yiannou P, et al (2011). MELF invasion in endometrial cancer as a risk factor for lymph node metastasis. Histopathology.

[6] Han G, Lim D, Leitao MM, Abu-Rustum NR, Soslow RA (2014). Histological features associated with occult lymph node metastasis in FIGO clinical stage I, grade I endometrioid carcinoma. Histopathology.

[7] Dogan Altunpulluk M, Kir G, Topal CS, Cetiner H, Gocmen A (2015). The association of the microcystic, elongated and fragmented (MELF) invasion pattern in endometrial carcinomas with deep myometrial invasion, lymphovascular space invasion and lymph node metastasis. J Obstet Gynaecol.

[8] Murray SK, Young RH, Scully RE (2003). Unusual epithelial and stromal changes in myoinvasive endometrioid adenocarcinomas: a study of their frequency, associated diagnostic problems, and prognostic significance. Int J Gynecol Pathol.

[9] Dabbs D (2013). Diagnostic Immunohistochemistry, Theranostic and Genomic Applications, Expert Consult, 4th ed.

[10] Euscher E, Fox P, Bassett R, Al-Ghawi H, Ali-Fehmi R, Barbuto D, et al (2013). The pattern of myometrial invasion as a predictor of lymph node metastasis or extrauterine disease in low-grade endometrial carcinoma. Am J Surg Pathol.

[11] Mutch DG (2009). The new FIGO staging system for cancers of the vulva, cervix, endometrium and sarcomas. Gynecol Oncol.

[12] Ali A, Black D, Soslow RA (2007). Difficulties in assessing the depth of myometrial invasion in endometrial carcinoma. Int J Gynecol Pathol.

[13] Hertel JD, Huettner PC, Pfeifer JD (2014). Lymphovascular space invasion in microcystic elongated and fragmented (MELF)-pattern well differentiated endometrioid adenocarcinoma is associated with a higher rate of lymph node metastasis. Int J Gynecol Pathol.

[14] Roma AA, Rybicki LA, Barbuto D, Euscher E, Djordjevic B, Frauenhoffer E, et al (2015). Risk factor analysis of recurrence in low-grade endometrial adenocarcinoma. Hum Pathol.

[15] Stewart CJ, Brennan BA, Leung YC, Little L (2009). MELF pattern invasion in endometrial carcinoma: association with low grade, myoinvasive endometrioid tumours, focal mucinous differentiation and vascular invasion. Pathology.

